# simpleaf: a simple, flexible, and scalable framework for single-cell data processing using alevin-fry

**DOI:** 10.1093/bioinformatics/btad614

**Published:** 2023-10-06

**Authors:** Dongze He, Rob Patro

**Affiliations:** Department of Cell Biology and Molecular Genetics and Center for Bioinformatics and Computational Biology, University of Maryland, College Park, MD, 20742, United States; Department of Computer Science and Center for Bioinformatics and Computational Biology, University of Maryland, College Park, MD, 20742, United States

## Abstract

**Summary:**

The alevin-fry ecosystem provides a robust and growing suite of programs for single-cell data processing. However, as new single-cell technologies are introduced, as the community continues to adjust best practices for data processing, and as the alevin-fry ecosystem itself expands and grows, it is becoming increasingly important to manage the complexity of alevin-fry’s single-cell preprocessing workflows while retaining the performance and flexibility that make these tools enticing. We introduce simpleaf, a program that simplifies the processing of single-cell data using tools from the alevin-fry ecosystem, and adds new functionality and capabilities, while retaining the flexibility and performance of the underlying tools.

**Availability and implementation:**

Simpleaf is written in Rust and released under a BSD 3-Clause license. It is freely available from its GitHub repository https://github.com/COMBINE-lab/simpleaf, and via bioconda. Documentation for simpleaf is available at https://simpleaf.readthedocs.io/en/latest/ and tutorials for simpleaf that have been developed can be accessed at https://combine-lab.github.io/alevin-fry-tutorials.

## 1 Introduction

Single-cell sequencing has become an indispensable tool for studying cellular biology at the resolution of individual cells (Stuart and Satija 2019), and processing the resulting data often requires a dedicated suite of tools and methods. Recently, He *et al.* (2022) demonstrated that the alevin-fry ecosystem provides an efficient, accurate, and flexible framework for single-cell data processing. Yet, the rapid arrival of new technologies and experimental modalities have led to data analysis pipelines that require increasingly complex and sophisticated workflows. For example, analyzing CITE-seq (Stoeckius *et al.* 2017) data involves executing the entire alevin-fry pipeline three times, each time with a slightly different configuration and on different sets of files. Likewise, as improved tools, like the piscem (Fan *et al.* 2022) read mapping tool, are introduced into the alevin-fry ecosystem, users wishing to adopt these new tools must learn their interfaces and logistics.

## 2 Software description

**Figure 1.**
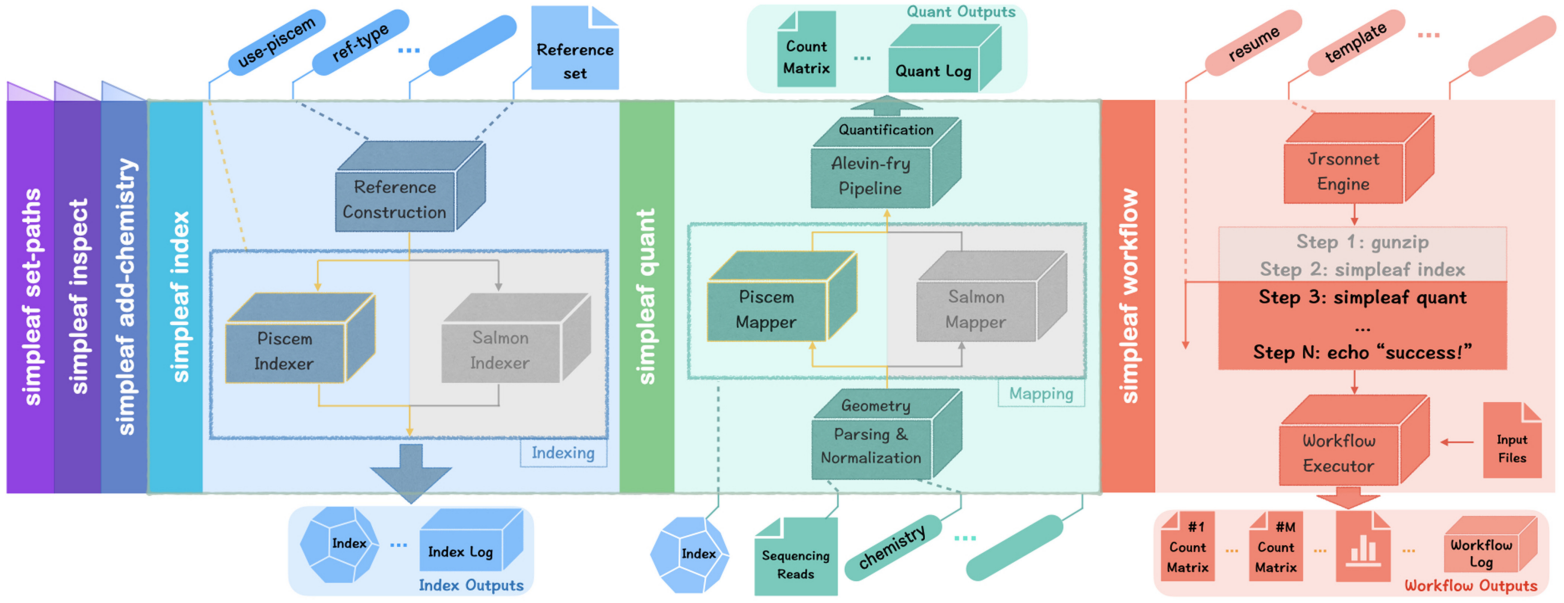
Overview of some salient simpleaf subcommands, showing the flow of data through a hypothetical invocation. The leftmost expanded column (blue) represents using the simpleaf index command to build a reference sequence and the corresponding piscem index. The center expanded column (aqua) represents using this piscem index in conjunction with sequenced reads to produce a count matrix for subsequent analysis. Note that the indexer and mapper of both piscem and salmon are fully supported in simpleaf. Finally, the rightmost expanded column (red) represents the invocation of a hypothetical simpleaf workflow, where the workflow can require several input files and produce several outputs. Additional simpleaf subcommands are described in the Supplementary Appendix

To simplify and ease the user experience for both simple and complex experimental setups, and to allow seamless use of the newest alevin-fry ecosystem components, we have developed simpleaf (simple alevin-fry). Simpleaf (overview in Fig. 1) is a high-level framework, that provides simple, flexible, and scalable interfaces for uniformly accessing standard and advanced features in the alevin-fry ecosystem.

The concept of “wrapper” or “workflow” programs in the context of single-cell data processing pipelines is well-established. Apart from the many bespoke workflows developed for individual technologies, there exist several tools designed to ease and simplify the processing of multiple types of data. Here, we highlight a few examples, though this is not intended, to constitute an exhaustive list of such tools. The popular Cell Ranger (Zheng *et al.* 2017) tool itself is, in part, a Python script that wraps STAR (Dobin *et al.* 2013) and other tools designed by 10× Genomics. The zUMIs (Parekh *et al.* 2018) and UniverSC (Battenberg *et al.* 2022) tools provide highly capable suites of workflows for processing data from many different technologies using, respectively, STAR and Cell Ranger itself. Kb-python is a Python tool that wraps kallisto|bustools (Melsted *et al.* 2021) and related tools, and provides a high-level interface to process data from many different experimental protocols and setups. The scPipe (Tian *et al.* 2018) tool is a modular wrapper around multiple tools and packages within the R ecosystem, which uses the Subread aligner (Liao *et al.* 2019) for mapping, and is capable of processing data from a variety of different single-cell technologies.

Simpleaf is dedicated to providing a simple and flexible user interface for the alevin-fry ecosystem, which consists of a range of underlying tools and modules for single-cell data processing (Patro *et al.* 2017, Srivastava *et al.* 2019, Khan and Patro 2021, Fan *et al.* 2022, He *et al.* 2022). In addition to coordinating the execution of these tools and providing a unified and simplified interface, simpleaf also adds new functionality aimed at further generalizing the capabilities of the underlying tools, and allowing users to easily create and share their own workflows without having to program or modify simpleaf itself.

### 2.1 A simplified interface to core alevin-fry functionality

The most basic functionality provided by simpleaf is that it provides a simplified yet flexible interface to the underlying alevin-fry modules and capabilities.

In simpleaf, the standard alevin-fry pipeline (He *et al.* 2022) is distributed over two phases: (i) indexing, which includes creating an augmented (*splici* (He *et al.* 2022) or *spliceu* (He *et al.* 2023)) reference, where appropriate, and building the corresponding reference index, and (ii) quantification, which consists of read mapping, cell barcode detection and correction, and UMI resolution. These two phases are exposed as two sub-commands within simpleaf: simpleaf index and simpleaf quant. Each of these, in turn, exposes various flags for retaining critical flexibility in processing.

Although most of the functionality provided by simpleaf programs can be directly replicated by calling the underlying tools with the appropriate configurations and arguments, the advantage of using simpleaf comes from the fact that simpleaf, by default, incorporates the best practices for running the underlying tools, reduces the workload by automatically handling tedious but crucial details one needs to take care of in the most common use cases, and also retains critical flexibility when necessary. For example, if a simpleaf index invocation is followed by a call to simpleaf quant, simpleaf quant will automatically recruit and parameterize the correct mapper, and will automatically locate and provide the file containing the transcript-to-gene mapping information to later quantification stages where appropriate. This file would have explicitly provided if alevin-fry is not invoked through simpleaf. Yet, to provide for maximum flexibility, simpleaf provides alternative processing options as well, like the option to begin the quantification process from an already-computed set of mapping results and thus to skip the mapping process.

### 2.2 Dedicated parameterization for easily switching between options and underlying tools

As the methodologies underlying single-cell quantification advance, we have continued to improve the existing features of the alevin-fry ecosystem and introduce new options and functionality wherever appropriate. Yet, the options and possibilities for single-cell analysis continue to expand, and the burden on users to keep up with new methods, tools, and best practices grows.

Simpleaf ensures that the best practices and new features of the alevin-fry ecosystem can be easily accessed and applied by users to their data. On one hand, every new version of simpleaf tracks and applies current best practices for the supported alevin-fry tools, and manages the relevant version requirements for the backing tools from the alevin-fry ecosystem. On the other hand, simpleaf provides simplified configurations for parameterizing its underlying tools. Especially for processing steps with multiple backend tool options available, simpleaf exposes a unified set of flags for controlling the find-grained parameterization of all relevant options, along with a flag for conveniently switching between options. As a result, users gain seamless access to all software tools in the alevin-fry ecosystem, including those alternatives, without the need to interact directly with each tool’s user interface. At the same time, simpleaf is designed to provide fine-grained access to the options and parameters of the underlying tools so that users *can* alter or modify these processing options and parameters if they wish.

One example is the ability to easily switch between piscem (Fan *et al.* 2022) and salmon (Patro *et al.* 2017, Srivastava *et al.* 2019) as the underlying reference indexing and mapping tools. Piscem is a new index and mapper in the alevin-fry ecosystem that further lowers the memory requirements for single-cell data processing (He *et al.* 2023), and salmon (Patro *et al.* 2017, Srivastava *et al.* 2019), which relies on the pufferfish index (Almodaresi *et al.* 2018), is the traditional mapper used with alevin-fry. As piscem and salmon are independent tools with distinct parameters, explicitly switching from salmon to piscem requires knowledge about the piscem tool and the relevant details of its indexing and mapping subcommands. However, in simpleaf, the only modification needed to make use of piscem is to pass the--use-piscem flag. Furthermore, for simplicity, if this flag is set when calling simpleaf index, the subsequent simpleaf quant executions that map against this index will automatically use piscem, with appropriate parameters, as the mapper.

Another example is the ability to seamlessly build different types of reference indices by simply changing the flags passed to the simpleaf index command. Currently, simpleaf has the ability to build three kinds of reference indices. Although the procedure for generating these types of reference indexes is different, simpleaf abstracts over the technicalities and only requires the user to set the--ref-type option as desired.

### 2.3 Parsing protocols with a complex fragment geometry

Another useful feature provided by simpleaf is the ability to represent and parse fragment geometry specifications that are potentially *more complex* than those directly supported by the underlying mappers—for example, those with variable barcode length and floating barcode position, such as sci-RNA-seq3 (Cao *et al.* 2019)—using a concise description language. (The current specification of this description language is available at https://hackmd.io/@PI7Og0l1ReeBZu_pjQGUQQ/rJMgmvr13.) An example of streaming parser invocation can be found in Supplementary Appendix Section SB.

When presented with a “complex” geometry specification, simpleaf “normalizes” these reads into an appropriate “simple” format (a format with only known position and fixed-length barcodes and UMIs) on the fly, and provides a modified (and simplified) geometry format description to the underlying mapper. Moreover, it streams the normalized reads directly to the mapper using FIFOs programmatically managed by simpleaf, thereby avoiding intermediate disk usage. This feature enables the existing mappers in the alevin-fry ecosystem, which are designed to process reads with simple geometry, to handle sophisticated geometries without modifying the underlying mapping tools, requiring extra preprocessing from the users, or taking the extra time and space to write and read the intermediate representations.

To date, the community has put significant efforts into documenting and categorizing the library layout for many existing sequencing assays (Battenberg *et al.* 2022, Booeshaghi *et al.* 2023). A good resource describing existing library layouts is the scg_lib_struct GitHub repository (https://github.com/Teichlab/scg_lib_structs). Furthermore, general parsers for such protocols have been developed (Smith *et al.* 2017, Liu 2019, Battenberg *et al.* 2022, Sullivan and Pachter 2023). Of course, these tools, or their relevant components could also be applied to this task, with the user handling the appropriate bookkeeping. However, the built-in capability of simpleaf focuses on providing a concise language for representing both simple and complex fragment geometry that can be passed directly to simpleaf from the command line, and the seamless internal normalization of this complex geometry into a simplified form compatible with both supported mappers.

### 2.4 Generalized and sharable workflow construction for complex single-cell workflows

Simpleaf also provides the ability to execute complex and highly configurable alevin-fry workflows described by simple user-provided configuration files. The purpose of the simpleaf workflow module is neither to replace general workflow languages like Nextflow (Tommaso *et al.* 2017) or Snakemake (Mölder *et al.* 2021) that enable near-limitless generality, but that require learning sophisticated and complex domain-specific languages, nor to expose some set of easy-to-use but pre-defined workflows for complex single-cell protocols as in Cell Ranger and kb-python. Rather, simpleaf workflow aims to provide a platform for alevin-fry users with all levels of programming knowledge to easily create, invoke, and share their workflows, which can contain not only simpleaf commands but also any shell commands that are valid in the user’s terminal. It allows the definition, via a simple imperative configuration and templating system, of custom workflows parameterized on user-defined input, which can then be reused to simplify the processing of complex workflows and easily shared with other users.

The simpleaf workflow module is built upon the idea of the simpleaf workflow template. Users can build their own template or, more commonly, obtain an existing (self-documented) template, fill in minimal information about the current sample to instantiate it, and execute the instantiated template using the workflow executor. More details can be found in Supplementary Appendix Sections SA.7 and SC. This design allows users with limited programming experience to define a simple but useful workflow as a JSON configuration, but also makes it possible for advanced users to develop sophisticated workflow templates to generate simpleaf workflows by taking advantage of the full functionality provided by the Jsonnet language. Furthermore, this design also makes it easy for users to create and share their workflows by simply sharing their workflow templates, without the need to understand and contribute to the codebase of simpleaf itself. To demonstrate the utility of the simpleaf workflow module, we have built such workflow templates for processing 10× Chromium 3′ v2 and v3 RNA-seq, CITE-seq (Stoeckius *et al.* 2017) and 10× feature barcode data. The list of currently published simpleaf workflow templates can be found in Supplementary AppendixTable S2. We expose the simpleaf workflow list program (Supplementary Appendix Section SA.9) for obtaining the list from the command line. We continue developing workflow configurations and are accepting contributions from the community.

## 3 Discussion

Simpleaf provides a simple and flexible interface to access the state-of-the-art features provided by the alevin-fry ecosystem, tracks best practices using the underlying tools, enables users to transparently process data with complex fragment geometry, and to build and execute sophisticated workflows containing both simpleaf and external commands without the need to write code. Simpleaf has already seen adoption in community-led projects, for example, in the scrna analysis pipeline of the nf-core project (Ewels *et al.* 2020, Peltzer *et al.* 2023). We hope that, in the future, simpleaf can serve as an entry point and main interface to the alevin-fry ecosystem for most users. We also envision that our workflow feature can encourage those in the community to create and share their workflows and, thus, can help simpleaf to provide increasingly reusable building blocks to enable more varied and sophisticated single-cell data analysis pipelines.

While simpleaf provides a simple and flexible framework for single-cell data processing, the current implementation still has limitations, which motivate future work. For example, although the fragment geometry parser can parse barcodes with variable length and floating position, it currently lacks the ability to perform certain kinds of preprocessing, like the barcode substitution scheme required by the split-seq (Rosenberg *et al.* 2018) technology. This can be solved by expanding the current geometry specification to describe and enable this kind of preprocessing. Moreover, simpleaf workflow is still under active development. Current efforts are underway to improve its generality, expand its library of standard functions, and develop more useful and sophisticated workflows for different purposes.

## Supplementary Material

btad614_Supplementary_Data
