# Supplementary material for: simpleaf: a simple, flexible, and scalable framework for single-cell data processing using alevin-fry
Source: Bioinformatics. 2023 Oct 6;39(10):btad614. doi: 10.1093/bioinformatics/btad614 (PMC10580267; doi:10.1093/bioinformatics/btad614)
Supplement: btad614_Supplementary_Data [file btad614_supplementary_data.pdf]

Appendices

Appendix A `simpleaf` subcommands

`Simpleaf` provides subcommands for various purposes. Here we briefly discuss the current subcommands exposed in `simpleaf`. The `alevin-fry` team is actively working to improve existing modules in the `alevin-fry` ecosystem as well as developing new modules that will be most useful for the community. For complete and up-to-date documentation, one can refer to the official `simpleaf` documentation at https://simpleaf.readthedocs.io/en/latest/. Table 1 lists all accessible tutorials that have been developed and released. New tutorials will be continuously developed and posted to the the alevin-fry tutorials library at https://combine-lab.github.io/alevin-fry-tutorials.

In order to operate properly, `simpleaf` requires that the environment variable `ALEVIN_FRY_HOME` exists. It will use the directory pointed to by this variable to cache useful information (e.g., the paths to selected versions of the tools it invokes, configurations containing the mappings for custom chemistries that have been registered, and other information like the permit lists for certain chemistries). In popular shells such as `bash` and `zsh`, this variable be set with `export ALEVIN_FRY_HOME=/full/path/to/dir/you/want/to/use`

A.1 `simpleaf set-paths`

Before calling `simpleaf`, one has to run the `simpleaf set-paths` command to set the paths to the underlying tools. If no flags are provided, this program will try to find the dependencies in the shell's `PATH`. Currently, to use `simpleaf`, one must provide a compatible version of `alevin-fry` (https://alevin-fry.readthedocs.io/en/latest/), and at least one of the `alevin-fry` mappers, `piscem` or `salmon`.

A.2 `simpleaf inspect`

This subcommand inspects the configuration of `simpleaf` in the current environment — such as the path to its dependencies and the custom chemistries that have been registered — and reports on the current configuration.

A.3 `simpleaf index`

The `simpleaf index` subcommand provides the functionality to build a reference index from the provided reference set using either the default `salmon` or the improved `piscem` indexing tool. Usually, for a species, protocol pair, `simpleaf index` needs only to be run once, and all subsequent experiments can utilize that index. By default, `simpleaf index` takes a reference genome in FASTA format and gene annotation in GTF format, and makes the *spliced+intronic* (*splici*) reference index after extracting the sequence of the spliced transcripts and intronic regions. Other augmented reference types, such as the *spliced+unspliced* (*spliceu*) reference, are also available in `simpleaf index` by setting the `--ref-type` argument appropriately.

If one would like to build the index directly from the provided reference sequences, for example, when the genome build of a species is unavailable and the transcriptome sequences are provided directly, one can pass the reference sequence file to the `--ref-seq` argument.

A.4 `simpleaf quant`

The `simpleaf quant` subcommand is designed as an all-in-one program to generate a gene×barcode count matrix directly from the provided index and sequencing reads. By default, it takes a `piscem` or `salmon` index, and the sequencing read (lists of `FASTQ` files) as input. It maps the reads and resolves the associated UMIs after detecting and correcting the cellular barcodes. It also provides the option to directly begin quantification from an already-computed set of mapping results, thereby skipping the mapping process, via the `--map-dir` argument.

A.5 `simpleaf add-chemistry`

This subcommand adds a new custom fragment geometry specification to the `simpleaf` geometry library together with a unique name. Once added, one can specify that custom geometry specification using the associated name when running `simpleaf quant`. For example, if one wants to store the geometry specification of sci-RNA-seq3, one can invoke `simpleaf add-chemistry --name sci-seq3 --geometry "1{b[9-10]f[CAGAGC]u[8]b[10]}2{r:}"`. Then, one can use this geometry when calling `simpleaf quant` by specifying `--chemistry sci-seq3`.

A.6 `simpleaf workflow get`

This subcommand gets the template of a registered workflow from a local copy of the protocol estuary GitHub repository (https://github.com/COMBINE-lab/protocol-estuary). If missing, `simpleaf` will automatically download the protocol estuary from GitHub into the `ALEVIN_FRY_HOME` directory. If invoking unpublished workflows, one can skip this step and provide the workflow templates directly to `simpleaf workflow` via the `--template` flag.

A.7 simpleaf workflow run

The `simpleaf workflow run` command is designed to run potentially complex single-cell data processing workflows according to an instantiated workflow template. During execution, the provided template in Jsonnet format will be first parsed into to a `simpleaf` workflow manifest in JSON format. Whereas the template provides logic and functions to handle features like variables and logical operations, the resulting workflow manifest is a simple imperative description of the commands that are to be executed.

Table 1. Current published simpleaf tutorials.

URL	Description
https://tinyurl.com/index-quant	This tutorial gives an example of processing a 10X Chromium v3 dataset from a provided set of FASTQ files natively or via simpleaf's Singularity container.
https://tinyurl.com/published-workflow	This tutorial introduces the flags exposed in the `simpleaf workflow run` program and shows the steps of processing a 10x feature barcode dataset through a readily available simpleaf workflow template.
https://tinyurl.com/custom-workflow	This tutorial elucidates the inner workings of the simpleaf workflow module and guides you through the step-by-step creation of a simpleaf workflow template for processing 10X Chromium v3 data from the ground up..

To ease the later parsing process, the values of all simpleaf flag arguments in any simpleaf template must be provided as strings, i.e., wrapped by quotes (`"value"`). `Simpleaf workflow` will traverse the resulting workflow manifest to find and parse the valid fields that record either a `simpleaf` or an external shell command. To provide the greatest flexibility, the only requirement `simpleaf workflow` sets is that the command record fields must follow a specific format.

1. A command record field must contain a `Step` and a `Program Name` sub-field, where the `Step` field represents which step, using an integer, this command constitutes in the workflow. The `Program Name` field represents a valid program in the user's execution environment. For example, the correct `Program Name` for `simpleaf index` is `"simpleaf index"` and for `simpleaf quant` is `"simpleaf quant"`. For an external command such as `awk`, if its binary is in the user's `PATH` environmental variable, it can just be `"awk"`; if not, it must contain a valid path to its binary, for example, `"/usr/bin/awk"`.
2. If a field records a `simpleaf` command, i.e., it has a valid `Step` and `Program Name` field, the name of the rest of its sub-fields must be valid `simpleaf` flags (for example, options like `--fasta`, or `-f` for short, for `simpleaf index` and `--unfiltered-pl`, or `-u` for short for `simpleaf quant`). Those option names (sub-field names), together with their values, if any, will be used to call the corresponding `simpleaf` program. Sub-fields that are not named by a valid `simpleaf` flag will trigger an error.
3. If a field records an external shell command, it must contain a `Step` and a `Program Name` sub-field as described above. In contrast to `simpleaf` command records, all arguments of an external shell command must be provided in an array, in order, with the name "`Arguments`". `Simpleaf workflow` will parse the entries in the array to build the actual command. For example, to tell `simpleaf workflow` to invoke the shell command `$ ls -l -h .` at step 7, one needs to use the following JSON record:

```
{
    "Step": 7,
    "Program Name": "ls",
    "Arguments": ["-l", "-h", "."]
}
```

After converting the workflow template into a `simpleaf` workflow manifest, `simpleaf workflow` will parse the commands in the workflow according to the following flags and put them into an execution queue. If none of the flags are set, `simpleaf workflow` will parse and invoke all commands recorded in the manifest except those that have a `Active` field, and this field is set as false.

- If setting the `--no-execution` flag, `simpleaf workflow` will parse the provided configuration file, write the log files, and return without any invocation.
- If setting the `--start-at` flag with a step number, `simpleaf workflow` ignore all previous steps and begin the invocation from that specific step (or the next active step if that specific step is inactive).
- If setting the `--resume` flag, `simpleaf workflow` will find the log file from a previous `simpleaf workflow` run in the provided output folder to decide which step to begin with.
- If setting the `--skip-step` flag with a set of comma-separated step numbers, `simpleaf workflow` will ignore the commands represented by those skipped step numbers during invocation.

Many useful and frequently used functions have been provided as a `simpleaf` workflow utility library. The documentation can be found at https://simpleaf.readthedocs.io/en/latest/workflow-utility-library.html.

A.8 `simpleaf workflow refresh`

This command pulls the latest protocol estuary from its GitHub repository (https://github.com/COMBINE-lab/protocol-estuary). We recommend updating the registry every time before pulling a workflow.

A.9 `simpleaf workflow list`

This command prints the lists of all workflows in the registry. We recommend invoking `simpleaf workflow refresh` every time before `simpleaf workflow list` to obtain the latest workflow template list. The simpleaf workflow templates currently published in the `protocol estuary` are listed in table 2.

Appendix B Example of streaming parser invocation

Internally, `simpleaf` will first check if the given geometry specification has a fixed barcode length and position. If it does, read files will be directly passed to the underlying mapper, designed to take fixed geometry reads. If not, `simpleaf` will pass the geometry specification to its sequence geometry parser (https://github.com/COMBINE-lab/seq_geom_parser) and call its sequence geometry transformer (https://github.com/COMBINE-lab/seq_geom_xform) to "normalize" the reads to a fixed geometry format and stream the transformed reads directly to the mapper, without writing intermediate files. For example, the simplified geometry description for the geometry originally specified in Appendix section A.5

Table 2. Current published simpleaf workflow templates. The list can also be obtained by invoking `simpleaf workflow list` command from the command line.

**Name**	**Target Data Type**
10x-chromium-3p-v2	10X Chromium v2
10x-chromium-3p-v3	10X Chromium v3
10x-feature-barcode-antibody	10X feature barcode experiment with antibody capture
10x-feature-barcode-crispr	10X feature barcode experiment with CRISPR
10x-feature-barcode-antibody+crispr	10X feature barcode experiment with antibody capture and CRISPR
cite-seq-ADT_10xv2	CITE-seq with ADT using 10X Chromium v2
cite-seq-ADT_10xv3	CITE-seq with ADT using 10X Chromium v3
cite-seq-ADT+HTO_10xv2	CITE-seq with both ADT and HTO using 10X Chromium v2
cite-seq-ADT+HTO_10xv3	CITE-seq with both ADT and HTO using 10X Chromium v3

is `1{b[11]u[8]b[10]}2{r:}`. The length of the first barcode changes from 9 or 10 nucleotides to 11 because the sequence geometry transformer augments the first barcode segment with an `A` if the original barcode is of length 10 or with `AC` if it is of length 9. By doing so, the first barcode of all reads will be of the same length, and the augmented barcodes of hairpin barcodes of different lengths will not overlap, as all hairpin barcodes of length 9 now end with `C` and all of the length 10 ends with `A` (this scheme can be naturally generalized to different segment width ranges).

Appendix C Example of simpleaf workflow invocation

As discussed in section A.7, **`simpleaf workflow`** is designed to generate and invoke single-cell analysis workflows according to a workflow template (Jsonnet program). **`Simpleaf`** uses the Jrsonnet (`https://github.com/CertainLach/jrsonnet`) library for parsing the underlying Jsonnet program.

In the protocol estuary GitHub repository (`https://github.com/COMBINE-lab/protocol-estuary`), we have published a workflow for processing CITE-seq data (Stoeckius et al., 2017). Once one has obtained the workflow template by calling **`simpleaf workflow get`** with the argument **`--name cite-seq_10xv2`** as discussed in Appendix section A.6 and has instantiated it by filling in all required fields, i.e., the file path to the needed files, they can call **`simpleaf workflow run`** and provide the instantiated template to the **`--template`** argument. **`simpleaf workflow`** will parse the template as a workflow manifest using Jrsonnet and recursively find and parse all valid command records in the workflow manifest.

Without the help of `simpleaf workflow`, one needs to develop and invoke 12 distinct commands in the shell, including preprocessing, to obtain all the desired results of this specific workflow. Using `simpleaf workflow`, one only needs to fill the path to the files of sequencing reads and reference sets in the configuration file, and pass it to **`simpleaf workflow run`** via the **`--template`** argument. Then, **`simpleaf workflow`** will automatically generate a data analysis pipeline for that specific dataset and invoke all required commands for analyzing the data.
